# Artificial intelligence and machine learning in immunosenescence: from biomarker discovery to clinical translation

**DOI:** 10.3389/fragi.2026.1851261

**Published:** 2026-08-06

**Authors:** Xi Chen, Yan Han, Ruixuan Zhang, Xinyang Huang, Jinwu Liu, Hanxu Xie, Ling Teng, Chen Han, Ziqi He, Zimeng Yang, Shihan Huang, Jianhui Yan

**Affiliations:** 1 Affiliated Hospital of Xiangnan University, Chenzhou, Hunan, China; 2 Clinical College of Xiangnan University, Chenzhou, Hunan, China; 3 Xiangnan University, Chenzhou, Hunan, China

**Keywords:** aging biomarkers, artificial intelligence, clinical translation, immunosenescence, machine learning

## Abstract

The global population is undergoing unprecedented aging, with immunosenescence established as a core upstream driver of nearly all age-related chronic diseases, imposing a massive global clinical burden. For decades, immunosenescence research has been mired in three persistent translational bottlenecks: inability to capture interindividual immune aging heterogeneity, failure to decode complex multi-layered biological regulatory networks, and inefficient therapeutic development pipelines. Artificial intelligence (AI) and machine learning (ML) have emerged as promising solutions to these bottlenecks, yet existing literature fails to systematically bridge AI technical advances with clinical immunology practice, and often does not distinguish proof-of-concept evidence from the steps needed for clinical use. This review provides a holistic, critical overview of AI/ML applications across the full translational spectrum of immunosenescence research, from mechanistic discovery, biomarker development, diagnostic innovation to therapeutic development and personalized medicine. We further analyze unresolved technical, ethical, and regulatory barriers to clinical translation, including underrecognized fundamental flaws in current model design. Emerging AI technologies to address these limitations are outlined, alongside a clinically realistic translational roadmap and key future research trends. This review fills critical gaps in existing literature, providing a rigorous framework to shift the field from “AI for AI’s sake” to clinical utility-focused research, ultimately advancing healthy aging interventions.

## 1 Introduction

The global population is undergoing an unprecedented demographic shift: by 2050, adults aged 65 years and older will surpass 1.6 billion, accounting for nearly 18% of the world’s total population (Barbé-Tuana et al., 2020). This graying of the global population has brought a tidal wave of age-related chronic diseases, now the leading cause of morbidity and mortality worldwide. Over the past 5 years, a growing body of work has cemented a central truth in geroscience: immunosenescenc—the progressive, highly heterogeneous decline of innate and adaptive immune function with age—is not just a byproduct of aging, but a core upstream driver of nearly all age-related chronic conditions in older adults (Barbé-Tuana et al., 2020; Conte et al., 2020). Its clinical reach extends far beyond infection susceptibility, driving malignant transformation, autoimmune dysregulation, neurodegeneration, frailty, and near-universal loss of vaccine efficacy in aging populations (Frasca and Blomberg, 2020; Li Y. et al., 2024; Yan et al., 2026).

For all its clinical importance, immunosenescence research has spent decades mired in three persistent translational bottlenecks. First, immune aging trajectories are highly variable across individuals, shaped by genetics, chronic viral exposure, psychosocial stress, metabolic comorbidities, gut dysbiosis, and environmental factors (Weltevrede et al., 2016; Aiello et al., 2016; Pallikkuth et al., 2021). Traditional low-dimensional analytical methods cannot capture this interindividual heterogeneity, nor define immune aging status in a way that is meaningful for individual patient care (Pawelec, 2020; Jia et al., 2023). Second, immunosenescence is governed by a multi-layered, interconnected biological network spanning cellular, molecular, and systemic levels, with constant crosstalk between the immune system, metabolic organs, gut microbiome, and central nervous system (Frasca and Blomberg, 2020; Yan et al., 2026; DeJong et al., 2020). Conventional methods lack the capacity to integrate high-dimensional multi-omics data needed to decode these networks, or disentangle correlation from causation (Bing et al., 2022; Zhang et al., 2024). Third, the translational pipeline for immunosenescence research is broken: there are almost no robust, clinically validated biomarkers in routine use, and over 90% of candidate senotherapeutic agents fail to replicate preclinical efficacy in human trials (Gadd et al., 2025; Nash et al., 2021; Wang et al., 2025). What the field needs is not more descriptive biology, but a more efficient, predictive research paradigm to bridge lab and clinic.

Artificial intelligence (AI) and machine learning (ML)—tools built for pattern recognition in high-dimensional complex data—have been put forward as a solution to these bottlenecks. In recent years, a small number of high-impact studies have shown real promise merging AI/ML with immunosenescence research: deep learning-derived immune aging clocks (iAge, PHARE model) have enabled more accurate biological age quantification and clinical outcome prediction (Zhang et al., 2024; Sayed et al., 2021); ML algorithms have identified novel causal regulators of immune aging and biomarkers for cancer immunotherapy response (Zhu et al., 2024; Chen et al., 2025); and AI-driven platforms have accelerated discovery and repurposing of senotherapeutic agents (Baker et al., 2025; Hou et al., 2025; Cao et al., 2025). The 2024 launch of the Immunosenescence Inventory, the first public multi-omics database curating standardized immune aging data, has also lowered barriers for AI-driven research, though its real-world impact remains unproven (Li et al., 2025).

Existing literature has critical gaps this review aims to address. Most reviews either focus on technical AI advances without clinical immunology grounding, or summarize immunosenescence biology without critically evaluating AI’s real clinical utility, failing to bridge the two fields. No comprehensive, up-to-date review systematically maps AI/ML applications across immunosenescence’s full translational pipeline—from mechanistic discovery to clinical implementation. Few address the technical, ethical, and regulatory barriers trapping AI-driven research in the lab, or provide a clinically realistic roadmap for future development. Most problematically, the vast majority of reviews perpetuate a trend of “AI for AI’s sake”: overstating model performance, ignoring fundamental biological limitations, and avoiding the reality that very few AI models in this field have moved beyond retrospective data analysis to prospective clinical use.

To clarify the scope of this review, we included studies in which AI or ML was used to measure, model, or target immune aging itself, rather than studies that only mentioned immunosenescence as background. We searched PubMed, Web of Science, and bioRxiv for work published from January 2019 to January 2026 using combinations of “immunosenescence,” “immune aging,” “artificial intelligence,” “machine learning,” and “deep learning,” and also checked references of relevant reviews and landmark papers.

We prioritized studies with measurable immune-aging features, such as age-related T- or B-cell remodeling, receptor repertoire changes, thymic output, senescence markers, SASP-related factors, inflammatory aging signatures, immune aging clocks, vaccine response, infection outcomes, frailty-related immune phenotypes, or immunosenescence-related therapeutic targets. Studies on oncology, infection, microbiome, neurodegeneration, or general aging were included only when immune-aging variables were directly analyzed. When adjacent AI-biomedical studies were cited only for methodological comparison, we identified them as analogous rather than direct immunosenescence evidence. Preprints and early computational reports were treated cautiously and were not used as central evidence for clinical translation.

**FIGURE 1:**
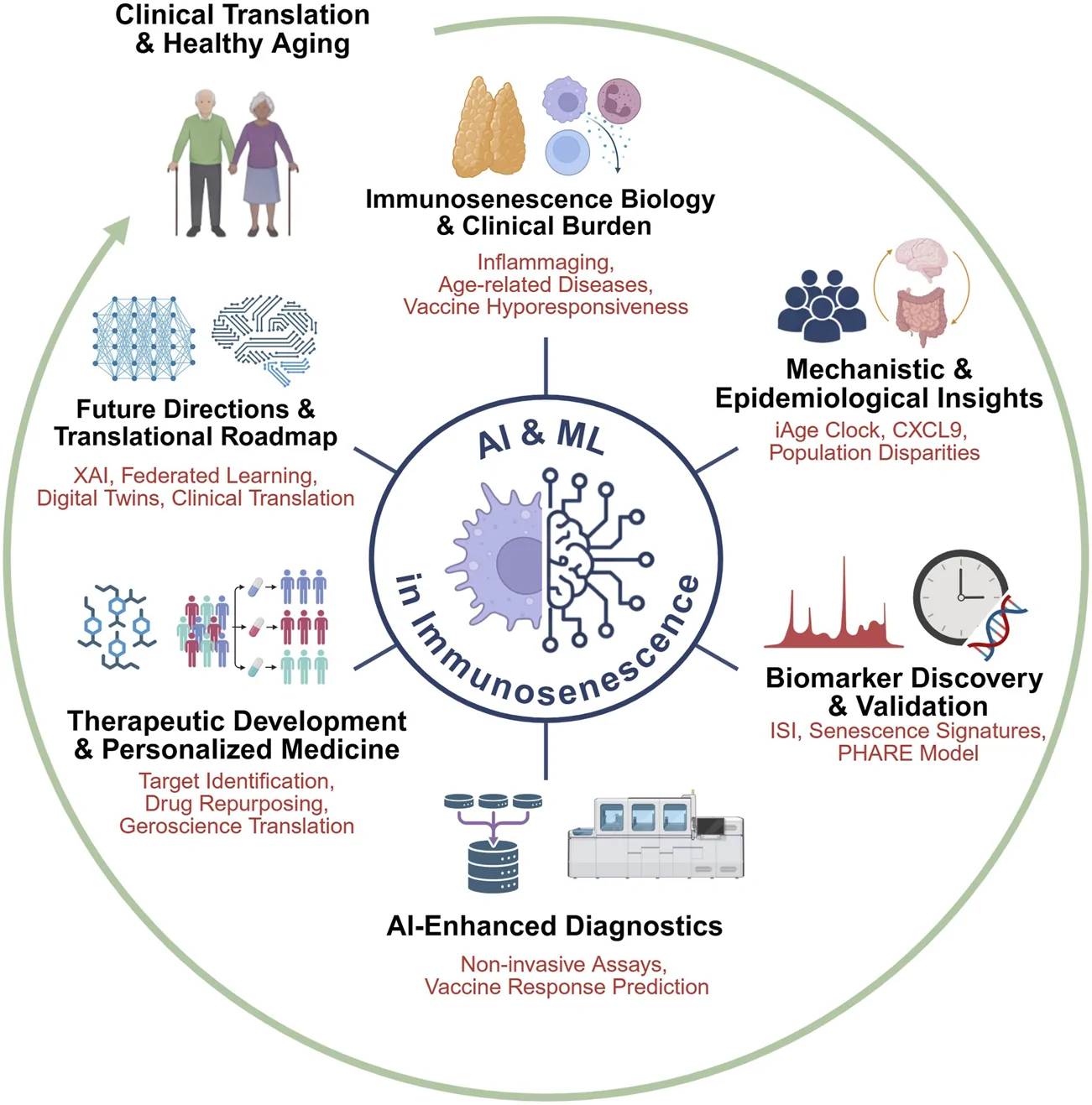
Graphical abstract of the review entitled Artificial Intelligence and Machine Learning in Immunosenescence: Mechanistic Insights, Clinical Translation, and Future Perspectives. This schematic illustrates the overarching framework of the review, positioning artificial intelligence (AI) and machine learning (ML) as tools to address the long-standing translational bottlenecks in immunosenescence research, mapping their applications across the full pipeline from basic biological discovery to clinical implementation.

In this review, we fill these gaps with a holistic, critical overview of the intersection of AI/ML and immunosenescence research (Figure 1). We first lay out the biological and clinical foundation of immunosenescence, including core mechanisms, clinical burden, and limitations of traditional methods that create a use case for AI/ML. We then break down core ML methodologies relevant to immune aging research (avoiding generic textbook overviews), and map key milestones in their integration with immunosenescence research over the past decade. Next, we synthesize AI/ML applications across the translational spectrum of immunosenescence, organizing each domain around the biological problem, the corresponding AI task, representative evidence, and remaining translational limitations. We also distinguish relatively well-validated examples from exploratory or forward-looking applications.

## 2 Biological and clinical foundation of immunosenescence

### 2.1 Core pathophysiological mechanisms of immunosenescence

Immunosenescence is driven by multiple interconnected, synergistic biological processes that collectively impair both innate and adaptive immune function. Recent single-cell and multi-omics studies have refined our understanding of these mechanisms, while making clear that immune aging is not a linear, uniform process, but a dynamic, multi-factorial syndrome with extensive systemic crosstalk (Conte et al., 2020; Zhang et al., 2024).

The most well-characterized initiating event of adaptive immune aging is age-related thymic involution, which begins in early adolescence and reduces naïve T cell output by over 90% by age 65 (Thomas et al., 2020). Progressive thymic function loss drives marked contraction of the T cell receptor (TCR) repertoire, initially shaped by V(D)J recombination under strict epigenetic regulation (Qiu et al., 2023). With age, disrupted epigenetic stability of TCR and immunoglobulin loci directly impairs V(D)J rearrangement efficiency, limiting *de novo* generation of diverse antigen receptors and further shrinking the adaptive immune repertoire (Jia et al., 2023; Thomas et al., 2020). The 2023 study by Jia et al—the largest healthy immune aging cohort to date (43,096 Chinese adults)—confirmed naïve CD4^+^ and CD8^+^ T cell decline is the most consistent age-related immune alteration across the lifespan, with significant sex-specific differences in T cell compartment aging rates (Jia et al., 2023).

Parallel to thymic involution, cellular senescence is a core driver of both innate and adaptive immune aging. Cellular senescence is defined as irreversible cell cycle arrest coupled with a bioactive senescence-associated secretory phenotype (SASP), including pro-inflammatory cytokines, chemokines, matrix metalloproteinases, and growth factors (Conte et al., 2020). Senescent cells accumulate in immune and non-immune stromal compartments with age, creating a self-reinforcing vicious cycle: SASP factors induce senescence in bystander immune cells, driving chronic low-grade systemic inflammation (*inflammaging*), which further exacerbates immune dysfunction and systemic aging (Conte et al., 2020; Frasca and Blomberg, 2020). A 2025 preclinical study by Gadd et al. confirmed senescent host hepatocytes directly impair systemic immune function and transplant engraftment in aged mice, expanding our view of immunosenescence beyond the immune compartment itself (Gadd et al., 2025). Critically, the 2021 iAge clock development identified CXCL9, a SASP-related chemokine, as a key causal mediator of both immunosenescence and cardiovascular aging, with *in vitro* and *in vivo* validation showing silencing CXCL9 reverses endothelial cell senescence and arterial stiffness (Sayed et al., 2021).

Metabolic dysfunction is another upstream causal driver of immunosenescence, with recent studies highlighting the critical role of immune cell metabolic rewiring in age-related functional decline (Conte et al., 2020). Mitochondrial dysfunction, impaired oxidative phosphorylation, and dysregulated glycolysis in aged immune cells directly impair cytokine production, proliferation, and effector function, with defects observed in both adaptive and innate compartments (Conte et al., 2020). Insulin resistance and hyperglycemia in type 2 diabetes further accelerate immune aging, increasing infection susceptibility and microvascular complication risk in older adults (Fernandes et al., 2019). It is worth noting, however, that most of these findings come from preclinical models, and the extent to which metabolic interventions can reverse human immune aging remains unproven in large clinical trials.

Finally, gut microbiota dysbiosis has emerged as a key systemic regulator of immunosenescence over the past 5 years, with studies establishing a causal gut-immune axis in aging. Age-related gut dysbiosis, characterized by reduced beneficial taxa (e.g., *Bifidobacterium*, butyrate-producing bacteria) and expanded pro-inflammatory species (e.g., Enterobacteriaceae*, Proteobacteria*), directly drives intestinal mucosal immune aging and systemic inflammaging (Pallikkuth et al., 2021; DeJong et al., 2020). A 2021 study in aged rhesus macaques by Pallikkuth et al. confirmed age-related gut dysbiosis directly correlates with elevated serum pro-inflammatory cytokines (IL-6, TNF-α) and systemic immune activation (Pallikkuth et al., 2021). Emerging evidence further links gut dysbiosis to central nervous system immunosenescence *via* the gut-brain axis, with microbiota-derived metabolites regulating microglial activation and neuroinflammation in Parkinson’s disease (PD) and Alzheimer’s disease (AD) (Yan et al., 2026; Haran et al., 2019). That said, most human studies in this space remain associative, with no consensus on which microbial taxa are causally linked to human immune aging, rather than just correlated with it.

### 2.2 Epidemiological and clinical burden of immunosenescence

The clinical consequences of immunosenescence are far-reaching, driving the majority of age-related morbidity and mortality worldwide, with growing epidemiological data quantifying its population-level impact and identifying historically overlooked high-risk subgroups (Barbé-Tuana et al., 2020; Moffa and Tana, 2025).

#### 2.2.1 Infectious diseases and vaccine hyporesponsiveness

Immunosenescence is the primary modifiable risk factor for severe infectious disease outcomes and mortality in older adults. Adults aged 65+ have 10–20 times higher COVID-19 mortality than younger individuals, with impaired viral clearance and dysregulated inflammatory responses driven by immunosenescence identified as the core causal mechanism in multiple large cohorts (Vellas et al., 2020; Cunha et al., 2020). Beyond respiratory viruses, herpes zoster (caused by varicella-zoster virus reactivation from impaired T cell immunity) affects one in three individuals over their lifetime, with 50% of vision-threatening ocular cases occurring exclusively in older adults with advanced immunosenescence (Minor and Payne, 2025). Immunosenescence also drives the growing multidrug-resistant organism (MDRO) infection crisis in aging populations: older adults with impaired immune function have a 3-fold higher risk of MDRO infection and infection-related mortality, with 2024 data showing immunosenescence markers directly predict antibiotic treatment failure in older adults (Moffa and Tana, 2025; Theodorakis et al., 2024).

A defining population-wide clinical consequence of immunosenescence is marked vaccine hyporesponsiveness in older adults. Influenza vaccine efficacy drops from 70% to 90% in young adults to just 30%–40% in adults aged 65+, with similar reductions observed for COVID-19, respiratory syncytial virus, and zoster vaccines (Bulut et al., 2020; Naserghandi et al., 2020). This loss of protection is directly driven by core immunosenescence hallmarks, including naïve T cell repertoire contraction, impaired T follicular helper cell function, and B cell senescence, creating a critical unmet clinical need for predictive biomarkers and optimized vaccine strategies for aging populations (Bulut et al., 2020; Gustafson et al., 2018; Bowler et al., 2022). Despite decades of research, there are still no FDA-approved biomarkers to predict vaccine response in older adults, a gap AI/ML has been proposed to fill.

#### 2.2.2 Malignancies

Immunosenescence is a core driver of cancer initiation, progression, and treatment resistance in older adults, who account for over 60% of new cancer diagnoses and 70% of cancer-related deaths worldwide. Age-related immune deterioration impairs malignant cell immune surveillance, with accumulated senescent immune cells and SASP factors creating an immunosuppressive tumor microenvironment that promotes oncogenesis and metastasis (Karakousis et al., 2020; Wu et al., 2025). Over the past 5 years, ML-driven studies have identified immunosenescence signatures that directly predict clinical outcomes in multiple malignancies: the machine learning-derived immunosenescence index (ISI) predicts survival and immune checkpoint inhibitor response in skin cutaneous melanoma, with low ISI correlating with favorable overall survival (HR = 0.56, p < 0.0001) and higher treatment response rates (Zhu et al., 2024). A 2025 study by Chen et al. developed and validated a T cell senescence-related gene signature that predicts prognosis and immunotherapeutic sensitivity in non-small cell lung cancer (NSCLC), with AUC values > 0.8 in two independent cohorts (Chen et al., 2025). It is important to note, however, that these signatures have not been validated in prospective clinical trials, and their utility in routine oncology care remains unproven.

#### 2.2.3 Autoimmune, neurodegenerative, and geriatric syndromes

Immunosenescence is tightly linked to age-related autoimmune disease pathogenesis, including elderly-onset rheumatoid arthritis (EORA) and psoriasis (Li Y. et al., 2024; Liu et al., 2024). EORA (onset after age 60) has a distinct clinical phenotype from younger-onset disease, with higher comorbidity burden and infection risk from immunosuppressive therapy, directly driven by underlying immune aging (Li Y. et al., 2024). A 2025 study further linked immunosenescence to difficult-to-treat rheumatoid arthritis, with clonal hematopoiesis of indeterminate potential (CHIP, a well-established hematopoietic aging marker) predicting treatment failure in older adults (Lehoczki et al., 2026).

In neurodegenerative diseases, immunosenescence and neuroinflammation are now recognized as core causal pathogenic drivers of PD and AD (Yan et al., 2026; Haran et al., 2019). A 2026 review by Yan et al. highlighted that age-related microglial senescence and impaired central nervous system immune surveillance drive α-synuclein pathology and dopaminergic neurodegeneration in PD, with immunosenescence markers directly predicting disease progression and cognitive decline (Yan et al., 2026). A 2024 epidemiological study further found accelerated immunosenescence drives earlier, steeper cognitive decline in American Indian/Alaskan Native adults, a population with marked dementia risk disparities historically excluded from aging research (Savin et al., 2024).

Finally, immunosenescence is a causal driver of frailty, a geriatric syndrome characterized by reduced physiological reserve and increased stress vulnerability. Elevated pro-inflammatory cytokines (IL-6, TNF-α) from inflammaging directly promote frailty progression, with immune aging markers predicting incident frailty and mortality in multiple longitudinal cohorts (Frasca and Blomberg, 2020; Barth et al., 2020). Adipose tissue, a key site of age-related senescent cell accumulation, has emerged as a critical link between immunosenescence and frailty, with SASP factors from senescent adipocytes driving systemic immune dysfunction and muscle wasting (Frasca and Blomberg, 2020).

### 2.3 Limitations of traditional methodologies in immunosenescence research

Despite significant advances in our understanding of immunosenescence biology over the past 2 decades, traditional research methodologies have critical unresolved limitations that have hindered translational progress, directly creating the urgent need for AI/ML-driven approaches.

First, traditional immune profiling methods are low-dimensional and fail to capture immune aging heterogeneity. For decades, immunosenescence research has relied on flow cytometry-based quantification of a small number of immune cell subsets (e.g., CD4:CD8 ratio, naïve vs. memory T cell frequency) and single cytokine measurements (Pawelec, 2020; Jia et al., 2023). While these methods have identified core immune aging hallmarks, they cannot resolve the cellular heterogeneity revealed by single-cell and spatial multi-omics technologies, nor accurately quantify immune aging status at the individual level (Jia et al., 2023; Zhang et al., 2024). For example, a 2022 study by Praja et al. found traditional partial least squares-discriminant analysis (PLS-DA) achieved only 64% accuracy in identifying older adults with high levels of pathogenic senescent CD4^+^ T cells, compared to 100% accuracy with a neural network model trained on the same dataset (Praja et al., 2022).

Second, conventional statistical approaches cannot integrate high-dimensional multi-omics data. Immunosenescence is regulated by a complex multi-layered network spanning the genome, epigenome, transcriptome, proteome, metabolome, and microbiome (DeJong et al., 2020; Bing et al., 2022). Traditional univariate and multivariate statistical methods are not designed to handle this extreme dimensionality, often leading to false positive findings, oversimplified causal inferences, and an inability to identify hidden regulatory patterns across biological layers (Bing et al., 2022). A 2022 study by Bing et al. demonstrated essential regression (ER), a latent-factor-regression-based ML approach, outperformed all conventional statistical methods in integrating multi-omic data to infer causal relationships in immunosenescence (Bing et al., 2022).

Third, traditional drug discovery and development pipelines for immunosenescence are inefficient and costly. Conventional target identification and lead compound optimization rely on low-throughput, hypothesis-driven experimental approaches, with a >90% attrition rate for candidate senotherapeutic agents in preclinical to clinical translation (Nash et al., 2021; Ansari et al., 2025). For example, senolytic agents have shown promising efficacy in preclinical models of immunosenescence, but few have demonstrated consistent benefits in large-scale human clinical trials (Gadd et al., 2025; Wang et al., 2025). AI-driven drug discovery platforms have emerged as a solution to this bottleneck, with deep learning models reducing the time and cost of lead compound identification by more than 80% in recent studies (Cao et al., 2025; Ansari et al., 2025).

Finally, traditional approaches lack the ability to predict individual immune aging trajectories and clinical outcomes. Most immunosenescence research to date has been cross-sectional, with conventional methods unable to model the dynamic, longitudinal nature of immune aging over the lifespan (Zhang et al., 2024). This has limited the development of personalized interventions, as clinicians cannot identify individuals at high risk of accelerated immune aging before clinical disease onset, nor predict which patients will respond to specific senotherapeutic or vaccine strategies (Pawelec, 2020; Pinto et al., 2025).

## 3 Fundamentals of AI/ML technologies and their integration with immunosenescence research

Building directly on the limitations of traditional methodologies outlined, this section outlines core AI/ML techniques with actual utility in immune aging research (avoiding generic textbook overviews), and maps key milestones in their integration with immunosenescence research over the past decade.

### 3.1 Core ML methodologies for immunosenescence research

ML algorithms used in immunosenescence research fall into three broad categories: supervised learning, unsupervised learning, and deep learning. Critically, not all ML methods are equally suited to immune aging research, and many studies have found simpler, more interpretable models often outperform complex deep learning approaches in real-world clinical settings, particularly with limited sample sizes. Each category has distinct strengths and well-defined use cases for addressing the core bottlenecks of immune aging research (Bing et al., 2022; Zhang et al., 2024).

Supervised learning algorithms are trained on labeled datasets to predict predefined outcomes, making them the most widely used tools for biomarker discovery, clinical outcome prediction, and diagnostic classification in immunosenescence research. Commonly used models include random forests, support vector machines (SVM), least absolute shrinkage and selection operator (LASSO) regression, and XGBoost (Jia et al., 2023; Liu et al., 2024). These models excel at feature selection from high-dimensional data, addressing the limitation of traditional methods in identifying robust immune aging markers from multi-omics datasets. For example, random forest models were used to identify naïve T-cell decline and memory T-cell expansion as key markers of immune aging in the 43,096-participant Chinese cohort (Jia et al., 2023). A key advantage of these models over deep learning is their relative interpretability, which is critical for clinical translation.

Unsupervised learning algorithms analyze unlabeled datasets to identify hidden patterns, clusters, and subpopulations, making them invaluable for resolving the interindividual heterogeneity of immune aging—a core limitation of traditional analytical approaches. Key techniques include hierarchical clustering, k-means clustering, and dimensionality reduction methods such as principal component analysis (PCA) and PLS-DA (Praja et al., 2022; Arvey et al., 2020). For example, unsupervised clustering of serum antibody binding profiles from 1,675 donors by Arvey et al. revealed age-dependent di-serine motif enrichment, aggregated into a longitudinally consistent “immune age” metric that correlated with autoimmune disease activity (Arvey et al., 2020). These methods are critical for identifying distinct immune aging subtypes and stratifying patient populations for personalized interventions, as they can uncover heterogeneity missed by hypothesis-driven, supervised approaches.

Deep learning is a subset of ML that uses multi-layered neural networks to process extremely high-dimensional data, including single-cell RNA sequencing (scRNA-seq), medical imaging, and multi-omics datasets, addressing the core challenge of integrating multi-modal data in immunosenescence research (Zhang et al., 2024; Praja et al., 2022). Convolutional neural networks (CNNs) are widely used for imaging analysis, while recurrent neural networks (RNNs) and transformer models excel at sequential and multi-modal data integration. Notable examples include the PHARE model, a deep learning tool trained on immune cell transcriptomes from newborns to supercentenarians that predicts biological age with high accuracy and identifies accelerated immune aging in pediatric COVID-19 patients with multisystem inflammatory syndrome (Zhang et al., 2024). Deep learning models have also achieved 100% accuracy in differentiating elderly individuals with high vs. low pathogenic CD4^+^ T-cell levels using serum ATR-FTIR spectroscopy data, outperforming traditional statistical methods by a significant margin (Praja et al., 2022). That said, deep learning models are far more prone to overfitting than simpler supervised learning approaches, particularly in small cohorts, and their “black box” nature remains a major barrier to clinical adoption.

### 3.2 Key milestones in the integration of AI/ML and immunosenescence research

**FIGURE 2:**
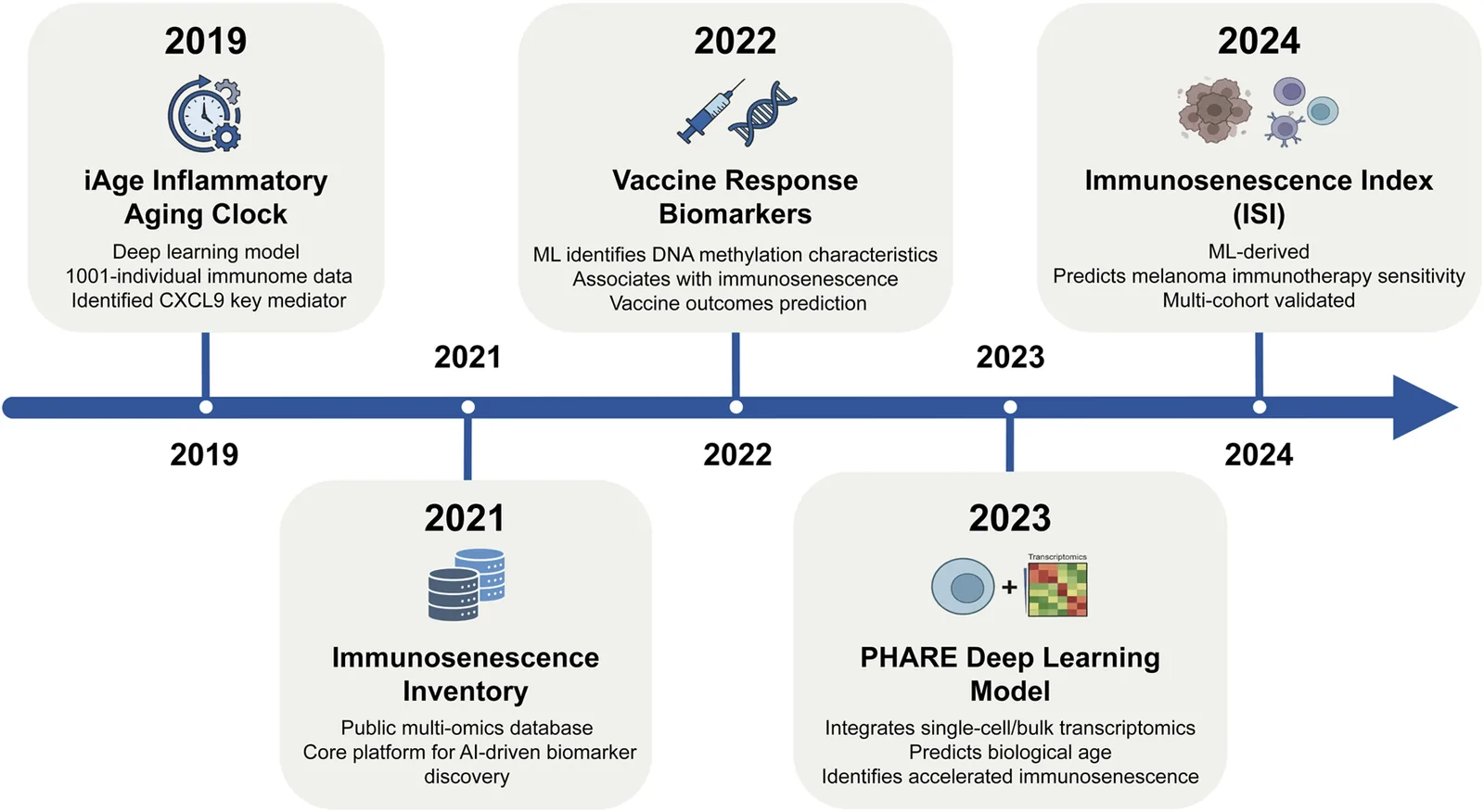
Chronological timeline of key landmark advances in the integration of artificial intelligence (AI) and machine learning (ML) with immunosenescence research. This timeline schematic systematically maps the foundational breakthroughs that have defined the convergence of AI/ML and immunosenescence research from 2019 to 2024.

The convergence of AI/ML and immunosenescence research has accelerated over the past decade, with a small number of landmark studies establishing the foundation of the field and marking a clear evolution from technical feasibility to clinical translation (Figure 2).

#### 3.2.1 2019: First deep learning-derived immune aging clock (iAge)

The development of the inflammatory aging clock (iAge), a deep learning model trained on blood immunome data from 1,001 individuals, marked the first major breakthrough in the field. iAge predicts multimorbidity, frailty, and cardiovascular aging with a mean absolute error of 7 years, and—critically—identified CXCL9 as a key causal mediator of immunosenescence and cardiac aging, with experimental validation in mouse and human models (Sayed et al., 2021). This study was the first to demonstrate that AI models could not only quantify immune aging, but also identify causal biological targets for intervention, rather than just correlative patterns.

The clock literature should also be read as a spectrum rather than a single model class. The longitudinal IMM-AGE metric used high-dimensional immune monitoring to define an immunological age trajectory that predicted survival beyond chronological age, while iAge emphasized inflammatory mediators and PHARE extended immune-age prediction to single-cell transcriptomic resolution (Zhang et al., 2024; Sayed et al., 2021; Alpert et al., 2019). These examples are relatively well-grounded immune-aging clocks, but they still differ in modality, endpoint, and clinical maturity. Future clock studies should therefore report whether the model predicts chronological age, an immune phenotype, or a patient-relevant outcome, because these are not interchangeable targets.

#### 3.2.2 2021: Launch of the immunosenescence inventory

The launch of the Immunosenescence Inventory, a public multi-omics database aggregating curated immune aging data from human studies, animal models, and clinical trials, provided a centralized resource for AI-driven analyses of immune aging. Prior to this, researchers were forced to use heterogeneous, unstandardized datasets from disparate sources, which limited model generalizability. This platform has become a foundational tool for biomarker discovery and therapeutic target identification, lowering barriers for AI research in the field (Li et al., 2025).

#### 3.2.3 2022: AI-driven prediction of vaccine responses in older adults

A landmark study used ML to identify DNA methylation biomarkers of influenza vaccine non-responsiveness in older adults, linking these epigenetic signatures to core immunosenescence hallmarks. This work established a framework for AI-driven pre-vaccination stratification of older adults, addressing a critical unmet clinical need in geriatric medicine (Bowler et al., 2022). While the model has not been validated prospectively, it provided a blueprint for how AI could be used to personalize vaccination strategies for aging populations.

#### 3.2.4 2023: Single-cell resolved deep learning immune aging model (PHARE)

Development of the PHARE deep learning model, which integrates single-cell and bulk transcriptomic data to predict biological age from immune cell gene expression profiles. This model enabled detection of accelerated immune aging in infectious and chronic diseases at single-cell resolution, overcoming the limitations of bulk tissue-based immune aging clocks that average out cellular heterogeneity (Zhang et al., 2024).

#### 3.2.5 2024: Retrospective clinical cohort validation of AI-derived immunosenescence biomarkers in oncology

Development of the machine learning-derived ISI, which predicts clinical outcomes and immunotherapy sensitivity in skin cutaneous melanoma, validated in multiple independent clinical cohorts. This study was the first to establish a paradigm for AI-driven immunosenescence biomarkers in potential clinical oncology workflows, moving beyond retrospective data analysis to a framework that could be adopted in clinical settings (Zhu et al., 2024).

## 4 Current applications and translational evidence of AI/ML in immunosenescence research

**FIGURE 3:**
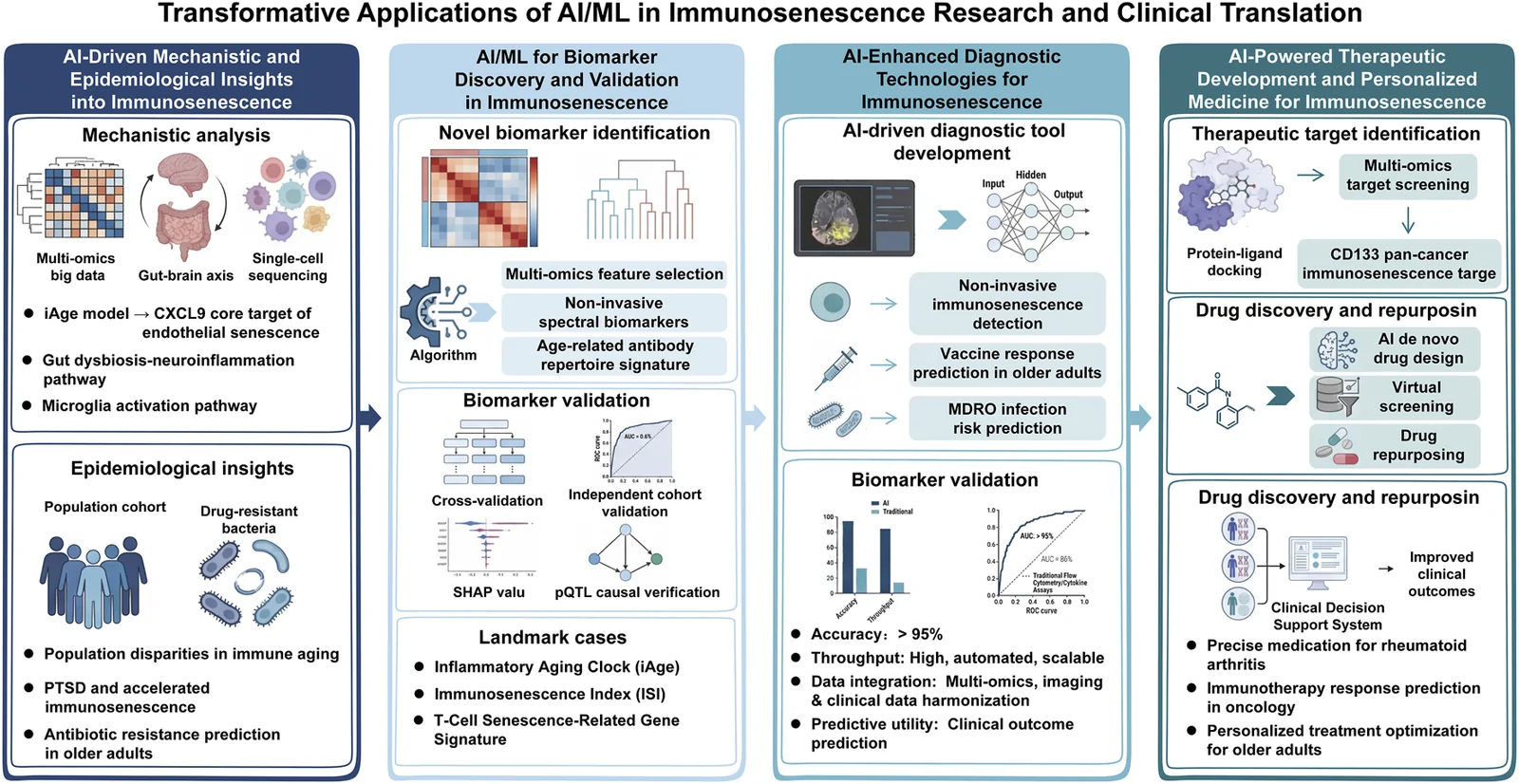
Current applications and translational evidence of artificial intelligence (AI) and machine learning (ML) in immunosenescence research and clinical translation. This comprehensive four-panel schematic outlines the full translational pipeline of AI/ML applications in immunosenescence research, organized into four interconnected domains: AI-driven mechanistic and epidemiological insights, AI/ML for biomarker discovery and validation, AI-enhanced diagnostic technologies, and AI-powered therapeutic development and personalized medicine. This framework demonstrates how AI/ML technologies can bridge basic biological insights to clinically actionable interventions for healthy aging, while highlighting the key barriers to translation at each stage.

Building on the technical foundation and landmark advances outlined above, this section synthesizes AI/ML applications across the translational pipeline of immunosenescence research (Figure 3). Most studies remain retrospective or proof-of-concept; only a small subset has undergone independent cohort validation, and almost none have been deployed as routine clinical tools.

We therefore distinguish direct immune-aging studies from disease-context applications and adjacent methodological examples. Direct studies measure, model, or target immune aging itself; disease-context examples are discussed only when immune-aging variables are directly analyzed. Adjacent AI-biomedical studies are used mainly to illustrate methods or possible workflows. In each subsection, we highlight the biological problem, AI task, representative evidence, and main translational limitation.

### 4.1 AI-driven mechanistic and epidemiological insights into immunosenescence

AI/ML has advanced our understanding of immunosenescence epidemiology and biology by integrating large-scale, multi-dimensional datasets to identify causal relationships and population-level disparities undetectable with traditional methods.

At the mechanistic level, AI models have decoded key regulatory pathways of immunosenescence previously hidden by the complexity of immune regulatory networks. The iAge deep learning model is the most prominent example: it not only quantified systemic immune aging, but also identified CXCL9 as a core driver of age-related endothelial senescence and arterial stiffness, with *in vitro* and *in vivo* validation (Sayed et al., 2021). In AD research, ML models integrated metagenomic and clinical data to establish a causal link between gut microbiota dysbiosis, impaired intestinal P-glycoprotein (P-gp) expression, and neuroinflammation, revealing a novel gut-brain axis mechanism of immunosenescence-related neurodegeneration (Haran et al., 2019). In PD, machine learning analysis of patient scRNA-seq data identified previously unrecognized microglial activation pathways and immunosenescence-related neuroinflammation mechanisms, guiding development of senolytic agents and T-cell immunotherapies for the disease (Yan et al., 2026).

Beyond single-omic analysis, ML-enabled multi-dimensional data integration has already been applied to decode immunosenescence regulatory networks. Three mainstream frameworks are currently used in practice: early fusion models that concatenate harmonized features across genomics, proteomics and metabolomics to build composite immune aging signatures; latent-factor approaches such as essential regression, which infer causal regulatory factors from multi-omic datasets and outperform conventional statistical methods in disentangling correlation from causation (Bing et al., 2022); and network-based models that map molecular interactions across biological layers. These integrative approaches overcome the limitations of siloed data analysis and provide a systems-level view of immune aging regulation, forming a methodological bridge from single-marker discovery to holistic clinical translation.

For the gut-immune axis in aging, AI-based causal inference and mediation analysis models have been applied to quantify the relationship between microbiota composition and immune aging phenotypes. Current studies integrate metagenomic sequencing data with immune cell profiling and systemic inflammatory markers to identify specific microbial taxa and metabolites associated with age-related immune decline, moving beyond simple correlational observations. For example, ML-driven mediation analysis can be used to estimate the proportion of inflammaging mediated by gut microbial dysbiosis, providing candidate targets for microbiota-directed immune aging interventions.

In epidemiological research, AI has enabled identification of population-level disparities and underrecognized risk factors for accelerated immunosenescence missed by traditional methods. Machine learning analysis of longitudinal cognitive and immune data by Savin et al. revealed American Indian/Alaskan Native adults experience earlier, steeper cognitive decline than non-Hispanic White individuals, driven by accelerated immunosenescence, highlighting the need for culturally tailored interventions for this high-risk population (Savin et al., 2024). AI models have also quantified the impact of psychosocial stress on immune aging, demonstrating post-traumatic stress disorder (PTSD) is associated with a 2.34-fold higher odds of late-differentiated effector CD8^+^ T-cell expansion and accelerated immune aging (Aiello et al., 2016). Additionally, ML algorithms integrate immunosenescence markers with clinical data to predict antibiotic resistance patterns in older adults, providing critical epidemiological insights to address the growing MDRO threat in aging populations (Theodorakis et al., 2024).

### 4.2 AI/ML for biomarker discovery and validation in immunosenescence

Development of robust, clinically useful biomarkers is the most urgent unmet need in immunosenescence research, and AI/ML has emerged as the most powerful tool for this purpose, addressing key limitations of traditional biomarker discovery approaches.

#### 4.2.1 AI-enabled identification of novel immunosenescence biomarkers

Supervised learning models excel at feature selection from high-dimensional multi-omics data, enabling identification of robust, reproducible immune aging biomarkers. Random forest models, LASSO regression, and XGBoost have been widely used to select predictive features from transcriptomic, proteomic, and flow cytometry datasets, identifying naïve T-cell decline, memory T-cell expansion, and cytokine signatures as key immune aging markers (Jia et al., 2023; Liu et al., 2024). Unsupervised learning approaches have identified novel biomarker candidates by resolving immune aging heterogeneity: clustering analysis of serum antibody repertoires identified an age-dependent di-serine motif signature that can be used as a biomarker of immune age and autoimmune disease activity (Arvey et al., 2020). Deep learning models have further expanded biomarker discovery to non-invasive modalities, with neural networks identifying spectral biomarkers of immunosenescence from serum ATR-FTIR spectroscopy data, enabling rapid, non-invasive assessment of immune aging status in clinical settings (Praja et al., 2022).

Aligning with the three major ML categories (supervised learning, unsupervised learning, deep learning) outlined in Section 3.1, different biomarker classes are typically paired with specific computational approaches based on data dimensionality, study purpose, and interpretability requirements. To make the biomarker-method link explicit, current AI/ML studies tend to emphasize different biomarker classes according to the computational task. Cytokine and SASP-related profiles, including IL-6, TNF-α, and CXCL9, are most naturally handled by supervised feature-selection models or inflammatory aging clocks when the endpoint is a clinical outcome such as frailty, vaccine response, or cardiovascular risk (Sayed et al., 2021; Bowler et al., 2022). TCR/BCR repertoire diversity and immune-cell subset remodeling are better suited to clustering, dimensionality reduction, or repertoire-similarity learning because their value often lies in detecting latent immune-age subtypes rather than a single marker (Jia et al., 2023; Arvey et al., 2020). Epigenetic clocks and DNA methylation signatures are commonly prioritized using regularized regression or ensemble models, whereas metabolic and microbiome signatures usually require multi-omics fusion or causal/network-based modeling to avoid treating correlated systemic changes as independent biomarkers (Pallikkuth et al., 2021; DeJong et al., 2020; Bing et al., 2022; Haran et al., 2019). This matching of biomarker class to model family is important because the most powerful tool is not necessarily the most clinically useful one.

That said, the field is plagued by a reproducibility crisis: hundreds of AI-derived immune aging biomarkers have been published in retrospective studies, but almost none have been validated in independent, diverse cohorts, let alone prospective clinical trials. This is a critical gap that has limited the clinical utility of these biomarkers.

#### 4.2.2 How AI/ML models prioritize immunosenescence biomarkers

Across the literature, ML prioritizes biomarkers through three related but distinct mechanisms. Supervised models rank features by their incremental contribution to a predefined outcome, such as vaccine non-responsiveness, frailty, survival, infection risk, or treatment response; this makes them most useful when the clinical endpoint is clear and the goal is a deployable risk model. Unsupervised models prioritize features that define stable clusters, trajectories, or latent immune-aging states, which is particularly valuable when the biology is heterogeneous and the outcome label is uncertain. Deep learning models can prioritize biomarkers through learned embeddings, attention weights, or *post hoc* explainability analyses, and are best suited to single-cell transcriptomics, spatial omics, spectroscopy, and other high-dimensional inputs, but they require larger datasets and stronger validation to avoid overfitting.

For clinical translation, biomarker ranking should therefore not be based only on statistical importance. A candidate biomarker should be stable across resampling and independent cohorts, add predictive value beyond chronological age and simple clinical variables, remain biologically plausible in immune-aging pathways, and be measurable using assays that can realistically enter clinical workflows. In this sense, interpretability, scalability, and translational readiness should determine tool preference: LASSO, Cox/logistic models, and random forests are often preferable for small-to-moderate clinical datasets; XGBoost or LightGBM can balance performance and feature ranking in larger tabular datasets; and deep learning should be reserved for settings where the data modality genuinely requires it and where explainability and external validation are built into model development.

#### 4.2.3 ML-driven rigorous validation of immunosenescence biomarkers

A major limitation of traditional immunosenescence biomarker research is the widespread lack of rigorous validation, with many candidate biomarkers failing to replicate in independent cohorts. ML models provide a robust framework for biomarker validation, with cross-validation, independent cohort testing, and causal inference methods ensuring the reliability and clinical utility of candidate markers (Radhakrishnan et al., 2024; Thelagathoti et al., 2025). For example, LASSO, XGBoost, and LightGBM models have been used to validate colorectal cancer proteomic biomarkers, with SHapley Additive exPlanations (SHAP) values and protein quantitative trait loci (pQTL) analysis confirming the biological relevance and causal role of the identified markers (Radhakrishnan et al., 2024). In gastric cancer research, random forest models validated a 10-biomarker panel of immunosenescence-related markers across multiple platforms, achieving an AUC of 0.952 in independent cohorts (Balikci and Kucukakcali, 2025). These ML-driven validation approaches directly address the reproducibility crisis in biomarker research, ensuring AI-identified markers are robust and generalizable to real-world patient populations—when implemented correctly.

#### 4.2.4 Representative successful cases of AI-Derived immunosenescence biomarkers

While most AI-derived biomarkers have failed to translate beyond retrospective analysis, a small number of successful cases have demonstrated real clinical utility, providing a blueprint for future biomarker development in the field.

Among the more mature direct immune-aging biomarkers, the most well-established is the iAge clock, a deep learning-derived biomarker that predicts multimorbidity, frailty, cardiovascular aging, and all-cause mortality, with CXCL9 identified as the core biological mediator driving its predictive performance. Notably, iAge has been validated in multiple independent cohorts, providing a robust, biologically interpretable metric of systemic immune aging that outperforms traditional chronological age-based risk stratification (Sayed et al., 2021).

Beyond systemic immune aging quantification, AI-derived immunosenescence biomarkers have been translated into oncology settings to guide clinical decision-making, exemplified by the machine learning-derived ISI. Developed and validated across multiple independent clinical cohorts, the ISI reliably predicts overall survival, intratumoral immune infiltration, and immune checkpoint inhibitor response in skin cutaneous melanoma, with low ISI correlating with significantly favorable overall survival (HR = 0.56, p < 0.0001) and higher treatment response rates (Zhu et al., 2024).

Further extending this oncology paradigm to other prevalent malignancies in aging populations, a machine learning-derived 3-gene T cell senescence-related signature (SLC2A1, TNS4, GGTLC1) has been developed and experimentally validated to predict prognosis and immunotherapeutic sensitivity in NSCLC, achieving AUC values > 0.8 in two independent cohorts, with *in vitro* studies confirming GGTLC1’s functional role in regulating T-cell senescence (Chen et al., 2025).

Collectively, these cases demonstrate AI-driven approaches can effectively translate high-dimensional immune aging data into clinically actionable biomarkers—when paired with rigorous biological validation and independent cohort testing. Unfortunately, these cases remain the exception rather than the rule in the field.

### 4.3 AI-enhanced diagnostic technologies for immunosenescence

AI has driven development of next-generation diagnostic tools for immunosenescence, addressing critical limitations of traditional diagnostic methods such as invasiveness, low throughput, and poor predictive accuracy.

#### 4.3.1 Development of AI-Driven diagnostic tools for immunosenescence

AI-enhanced diagnostic tools integrate multi-modal data (spectroscopy, omics, imaging, clinical parameters) to enable non-invasive, high-throughput, accurate assessment of immunosenescence status. A notable example is the combination of ATR-FTIR spectroscopy with neural networks, which achieves 100% accuracy in differentiating elderly individuals with high vs. low levels of pathogenic CD4^+^ T cells (a key immunosenescence marker) using only serum samples (Praja et al., 2022). This tool provides a non-invasive, rapid alternative to traditional flow cytometry-based immune profiling, which is labor-intensive and requires specialized laboratory infrastructure. The Immunosenescence Inventory database further supports diagnostic tool development by providing a centralized, curated resource for AI model training and validation across diverse patient populations (Li et al., 2025).

In clinical settings, AI models have been developed to predict functional outcomes of immunosenescence, including vaccine non-responsiveness and antibiotic resistance in older adults. ML models integrate immunosenescence markers with clinical data to predict influenza and COVID-19 vaccine responses in older adults, enabling pre-vaccination identification of non-responders and guiding personalized immunization strategies (Bowler et al., 2022). Deep learning models also predict MDRO resistance patterns in older adults, integrating immune aging markers to personalize antibiotic therapy and reduce treatment failure risk (Theodorakis et al., 2024).

#### 4.3.2 Performance comparison: AI-based vs. traditional diagnostic methods

AI-based diagnostic methods offer substantial advantages over traditional approaches for immunosenescence assessment, as summarized in Table 1. Traditional methods (flow cytometry, cytokine assays) are labor-intensive, time-consuming, and often lack sensitivity to detect early-stage immune aging (Theodorakis et al., 2024; Khalid et al., 2024). In contrast, AI-based methods provide high throughput, superior accuracy, and non-invasiveness. For example, neural network analysis of ATR-FTIR spectra achieves 100% accuracy in detecting pathogenic CD4^+^ T-cell expansion, compared to 64% accuracy with traditional PLS-DA analysis (Praja et al., 2022).

**TABLE 1:** Comparison of traditional and AI-based diagnostic methods for immunosenescence.

Feature	Traditional diagnostic methods	AI-based diagnostic methods
Accuracy	Moderate (60%–70% for most assays)	High (up to 100% in validated models) (Praja et al., 2022)
Throughput	Low to medium, labor-intensive	High, automated, scalable
Invasiveness	Often requires invasive sample collection (e.g., PBMC isolation)	Enables non-invasive assessment (e.g., serum spectroscopy) (Praja et al., 2022)
Multi-modal data integration	Limited to low-dimensional parameter analysis	Seamlessly integrates multi-omics, imaging, and clinical data (Li Y. et al., 2024)
Predictive utility	Primarily descriptive, limited prognostic value	Enables prediction of clinical outcomes (e.g., vaccine response, disease risk) (Bowler et al., 2022)

AI-based methods also excel at integrating multi-modal data, but several examples cited here are adjacent-field analogies rather than direct immunosenescence tools. For instance, a multimodal transformer integrating chest X-ray images and clinical text achieved an AUC of 0.912 for pediatric pneumonia severity assessment, and AI-enhanced endoscopy shortened diagnostic delay in laryngeal cancer while improving sensitivity (Li J. et al., 2024; Hu et al., 2025). These studies illustrate how multi-modal AI can outperform traditional workflows, but they should not be read as evidence that an immune-aging diagnostic tool is already clinically validated. At present, no AI diagnostic model has been specifically validated or approved for routine immunosenescence assessment.

### 4.4 AI-powered therapeutic development and personalized medicine for immunosenescence

AI/ML is transforming the development of immunosenescence-targeted therapies, from target identification and drug discovery to personalized treatment implementation, addressing the low efficiency and high attrition rates of traditional therapeutic development pipelines.

#### 4.4.1 AI-driven therapeutic target identification for immunosenescence

AI models accelerate therapeutic target identification by integrating multi-omics data, protein structure predictions, and biological network analysis to identify and prioritize key regulators of immunosenescence. Deep learning models predict protein-ligand interactions with high accuracy, enabling identification of novel molecular targets for immune aging intervention (Ansari et al., 2025; Catacutan et al., 2025). In oncology, transformer-based deep learning models identified CD133 as a core immunosenescence-related pan-cancer target, and screened a Chinese herb compound library to identify Polyphyllin V and Polyphyllin H as CD133-targeting agents, with experimental validation confirming their anti-tumor efficacy *in vitro* and *in vivo* (Hou et al., 2025). In neurodegenerative diseases, machine learning analysis of PD patient scRNA-seq data identified microglial activation pathways and immunosenescence-related targets, guiding senolytic agent development for the disease (Yan et al., 2026). These applications demonstrate AI can rapidly identify and prioritize immunosenescence therapeutic targets, drastically reducing the time and cost of preclinical target validation.

#### 4.4.2 ML-accelerated drug discovery and repurposing for immunosenescence

ML has accelerated virtual screening, drug repurposing, and rational design for immunosenescence-related conditions, although most examples remain preclinical. For example, the ApexAmphion platform uses reinforcement learning and protein language models for antimicrobial peptide design, but this report is currently a bioRxiv preprint and should be treated only as a forward-looking example rather than established evidence for immunosenescence therapy (Cao et al., 2025). AI-driven virtual screening has also identified candidate compounds in immune-aging-related disease contexts, including psoriasis and antibiotic resistance, but these findings still require peer-reviewed replication, mechanistic validation, and human testing before clinical use (Theodorakis et al., 2024; Tar et al., 2025; Tian et al., 2025).

#### 4.4.3 AI-enabled personalized medicine for immunosenescence-related diseases

Immunosenescence is highly heterogeneous, with substantial interindividual variability in therapeutic response and adverse event risk, making personalized medicine essential for effective intervention. AI models integrate immunosenescence biomarkers, genetic background, comorbidities, and clinical data to tailor treatments to individual patients, improving efficacy and reducing adverse events (Li Y. et al., 2024; Lehoczki et al., 2026).

In EORA, AI-assisted clinical decision support systems integrate gene expression profiling and immunosenescence biomarker analysis to optimize biologic therapy selection: IL-6 inhibitors are prioritized for patients with high inflammatory burden, while JAK inhibitors are recommended for those with cardiovascular comorbidities, addressing the increased infection risk associated with long-term TNF-α inhibitor use in older adults (Li Y. et al., 2024). In difficult-to-treat rheumatoid arthritis, ML models predict treatment failure risk by analyzing immunosenescence markers and CHIP status, guiding targeted therapy selection for high-risk patients (Lehoczki et al., 2026). In oncology, the ISI biomarker predicts immune checkpoint inhibitor response in skin cutaneous melanoma, with low ISI correlating with higher immune infiltration and better treatment outcomes, enabling patient stratification for personalized immunotherapy (Zhu et al., 2024). These applications establish AI as a powerful tool for translating immunosenescence research into personalized clinical care, addressing the unmet need for tailored interventions for older adults (Table 2).

**TABLE 2:** Summary of key AI/ML studies and resources in immunosenescence research.

Study (Ref)	Cohort size/context	Modality	Endpoint/AI task	Validation type	Evidence level/translational status
iAge clock (Sayed et al., 2021)	1,001 in primary cohort; additional validation cohorts	Blood immunome/inflammatory mediators	Immune age, multimorbidity, frailty, cardiovascular aging	Independent cohorts; experimental validation of CXCL9	Retrospective with independent and experimental validation; not a deployed test
PHARE model (Zhang et al., 2024)	Multi-age cohorts from newborns to supercentenarians	scRNA-seq and bulk immune-cell transcriptomics	Biological age and accelerated immune aging	Cross-dataset validation	Retrospective model validation; no prospective clinical trial
ISI in SKCM (Zhu et al., 2024)	Multiple retrospective melanoma cohorts	Transcriptomics and clinical data	Overall survival, immune infiltration, ICI response	Independent retrospective cohorts	Disease-context immune-aging biomarker; not routine care
T-cell senescence NSCLC signature (Chen et al., 2025)	Two NSCLC cohorts with experimental follow-up	Transcriptomic signature	Prognosis and immunotherapy sensitivity	Independent cohorts; *in vitro* validation	Disease-context biomarker with experimental support; no prospective deployment
ATR-FTIR neural-network model (Praja et al., 2022)	Older adult serum cohort	Serum ATR-FTIR spectroscopy	High vs. low pathogenic CD4^+^ T-cell burden	Internal validation and comparison with PLS-DA	Direct diagnostic prototype; external validation needed
Vaccine-response ML example (Bowler et al., 2022)	Older adult vaccination context	Epigenetic/immune-response features	Influenza vaccine non-responsiveness	Single-cohort analysis	Retrospective proof-of-concept; needs prospective validation
Antibody repertoire clustering (Arvey et al., 2020)	1,675 donors	Serum antibody binding profiles	Immune-age metric and autoimmune activity	Longitudinal consistency analysis	Retrospective with longitudinal support
Immunosenescence Inventory (Li et al., 2025)	Aggregated multi-study resource	Curated immune-aging multi-omics data	Database for AI model training and hypothesis generation	Not applicable	Infrastructure resource; real-world impact not yet proven

## 5 Challenges and barriers to clinical translation of AI/ML in immunosenescence

Despite the promising advances outlined in the previous section, the reality is that almost no AI/ML tools for immunosenescence have made it into routine clinical care. Multiple technical, ethical, regulatory, and clinical barriers remain, and addressing them is essential if the field is to move beyond “AI for AI’s sake” research into real patient benefit.

### 5.1 Technical and biological limitations

The most fundamental barrier to clinical translation is the lack of reproducibility and generalizability of AI models in immunosenescence research. The vast majority of AI models for immunosenescence are trained on single-center, homogeneous cohorts, and fail to replicate in independent, diverse populations (Chen et al., 2023). Overfitting is a pervasive issue: models often perform near-perfectly on training data but collapse when applied to real-world data, driven by the high dimensionality of omics data and small sample sizes of most immunosenescence studies. This problem is exacerbated by the fact that frail older adults with multiple comorbidities—who bear the highest burden of immunosenescence-related disease—are almost universally excluded from research cohorts, meaning models are trained on healthy older adults and fail when applied to the patients who need them most.

Across the studies discussed here, model rigor should be judged by more than headline AUC or accuracy. At minimum, reliable AI/ML immunosenescence studies should show adequate sample size relative to feature dimensionality, held-out testing and ideally external validation, calibration, batch-effect correction for omics or multi-center data, handling of class imbalance, adjustment for plausible confounders such as sex, comorbidity, BMI, medication exposure, ancestry, and CMV status when available, and comparison with simpler baselines such as logistic regression, Cox regression, or clinical scoring systems. Studies missing most of these safeguards should be considered exploratory even when their reported performance is high.

A largely unaddressed, fundamental flaw in the field is the widespread use of chronological age as the ground truth for AI model training. While chronological age is a convenient, easy-to-obtain label, it is a poor proxy for biological immune aging, as it does not account for dramatic interindividual heterogeneity in immune aging rates. Many state-of-the-art AI models, including the widely cited iAge clock, are trained to predict chronological age, not biological aging or clinical outcomes. This leads models to learn correlates of chronological age, rather than the causal drivers of immune senescence. While iAge was able to identify CXCL9 as a causal mediator despite this training paradigm, it is the exception rather than the rule: most chronological age-trained models identify features that correlate with age but have no causal role in immune aging, making them useless as therapeutic targets or clinical biomarkers. This fundamental design flaw has led the field down a path of building increasingly complex models that predict a label with no clinical relevance, a gap that has been almost entirely ignored in existing reviews.

To move beyond this limitation, future models should be trained against endpoints closer to immune function. Practical labels include frailty onset or progression, vaccine seroconversion or T-cell recall responses, infection frequency, severity, or pathogen clearance during follow-up, and composite immune-age phenotypes combining TCR diversity, CD4:CD8 ratio, naive/memory T-cell balance, inflammatory markers such as IL-6 and CXCL9, and functional assays. Chronological age can remain a covariate or benchmark, but not the default ground truth for clinical risk stratification or target discovery.

The “black box” nature of many AI models, particularly deep learning models, is another critical barrier to clinical translation (Oyewole-Said et al., 2020). Most state-of-the-art models lack interpretability: researchers and clinicians cannot explain *why* a model makes a given prediction, only that it does. This limits their utility for mechanistic research, as they cannot be used to derive causal biological insights, and erodes clinician trust in AI-driven clinical decisions. A clinician cannot justify a treatment recommendation to a patient based on an opaque model output, no matter how high the reported AUC is. While explainable AI (XAI) techniques such as SHAP and LIME are being developed to address this issue, their integration into immunosenescence research remains extremely limited, with most studies focusing solely on model performance metrics rather than interpretability (Celepli et al., 2025).

Biologically, the dynamic, longitudinal nature of immunosenescence poses a major challenge for static AI models. Almost all current models are trained on cross-sectional data, providing a single snapshot in time, and fail to capture the longitudinal trajectories of immune aging over the lifespan. Additionally, the complex crosstalk between immunosenescence and other aging processes, comorbidities, and environmental exposures creates substantial heterogeneity that is not fully captured by existing models, limiting their predictive accuracy in real-world clinical populations (Pinto et al., 2025).

### 5.2 Data-related ethical challenges

Data privacy is a paramount ethical concern in AI-driven immunosenescence research. Multi-omics data, electronic health records (EHRs), and imaging data used to train AI models contain highly sensitive personal health information, creating significant risks of data breaches and misuse (Shah et al., 2025). While public databases such as the Immunosenescence Inventory facilitate research, they must comply with strict global regulations including GDPR and HIPAA to protect patient privacy, creating significant administrative barriers to data sharing and multi-center research (Li et al., 2025). This has led to a siloed research landscape, where most groups work with their own small, single-center datasets, limiting model generalizability.

Data bias and lack of population diversity are critical ethical and technical challenges that exacerbate health disparities. Most AI models for immunosenescence are trained on data from healthy, highly educated, predominantly White populations, with severe underrepresentation of frail older adults, racial and ethnic minorities, and individuals with multiple comorbidities (Pinto et al., 2025). This leads to models that perform well in the population they were trained on, but have reduced accuracy or even cause harm in underrepresented populations, exacerbating existing health disparities in aging care. Informed consent is another ethical challenge, particularly for older adults with cognitive impairment, who may lack the capacity to provide fully informed consent for the use of their data in AI model training (Ozmen and Taub, 2026).

### 5.3 Regulatory and clinical translation barriers

Regulatory oversight for AI/ML-based software as a medical device (SaMD) is rapidly evolving, but remains a major barrier to clinical translation. The US FDA classifies AI-driven diagnostic and therapeutic decision support tools based on risk, with high-risk devices requiring pre-market approval (PMA). This entails rigorous analytical and clinical validation in large, diverse cohorts—something that almost no AI models for immunosenescence have completed (Shah et al., 2025). Without this large-scale, multi-center clinical validation, these models will never receive regulatory approval, no matter how well they perform in retrospective studies.

A unique challenge for AI-driven SaMD is model adaptability and post-market surveillance. Unlike traditional medical devices, many AI models are designed to continuously learn and update with real-world data, which creates significant regulatory challenges for ensuring consistent performance and safety over time (Ozmen and Taub, 2026). The FDA’s SaMD Action Plan requires manufacturers to implement rigorous post-market surveillance to monitor for model performance drift and bias, but almost no AI models for immunosenescence have established post-market monitoring frameworks (Shah et al., 2025).

Finally, even if these regulatory hurdles are overcome, significant clinical implementation barriers remain. Many healthcare systems, particularly those serving older adult populations in resource-limited settings, lack the digital infrastructure to integrate AI models into clinical workflows. Even in well-resourced systems, many clinicians lack the training to interpret and act on AI-driven insights for immunosenescence, a field that is already highly specialized. Most critically, the clinical utility of almost all AI models in this field remains unproven: while many models show excellent performance in retrospective analyses, almost none have been validated in prospective clinical trials to demonstrate that they actually improve patient outcomes, rather than just predict them (Nash et al., 2021).

## 6 Future directions and translational roadmap

Building directly on the challenges and barriers to clinical translation outlined, this section outlines emerging AI technologies that address these limitations, key future research trends, and a realistic, iterative clinical translation roadmap for AI-driven immunosenescence research. Unlike idealized linear pipelines presented in most reviews, this roadmap acknowledges the iterative, often unpredictable nature of translational research, and centers on clinical utility rather than model complexity.

### 6.1 Emerging AI technologies to address current limitations

A suite of innovative AI technologies has emerged to directly overcome the core technical, ethical, and translational barriers identified in immunosenescence research, offering targeted solutions to the most pressing limitations of current models, summarized in Table 3.

**TABLE 3:** Emerging AI technologies and integrative modeling frameworks for addressing core limitations in immunosenescence research.

AI technology/modeling framework	Core implementation details	Core barriers and limitations addressed	References
Explainable AI (XAI)	• SHAP/LIME-based model explanation • Attention-based feature interpretation • Mechanism-oriented biomarker decoding	• “Black box” opacity of AI models • Limited biological interpretability • Low clinician acceptance	Celepli et al. (2025)
Federated learning	• Distributed multi-center training • Raw data-free collaboration • Privacy-preserving model development	• Data privacy risks • Multi-center data sharing barriers • Poor generalizability from homogeneous cohorts	Ma et al. (2025)
Multi-omics fusion models	• Early, late, and intermediate fusion • Autoencoder-based latent representation • Multi-modal immune-aging signature construction	• Fragmented single-omics analysis • Limited multi-layer network integration • Missingness and batch-effect challenges	Bing et al. (2022), Zhang et al. (2024), Li et al. (2025)
Bayesian and causal inference models	• Prior knowledge-guided modeling • Uncertainty estimation • Causal and mediation analysis	• Correlation-dominated biomarker discovery • Weak causal inference • Poor uncertainty handling	Pallikkuth et al. (2021), DeJong et al. (2020), Bing et al. (2022), Haran et al. (2019)
Graph neural networks (GNNs) and network-based modeling	• Immune network representation • Cell–cytokine–metabolite interaction modeling • Spatial neighborhood analysis	• Limited biological interaction modeling • Poor spatial microenvironment representation • Incomplete systems-level immune-aging mapping	Hu et al. (2021), Dong and Zhang (2022), Ma et al. (2024)
Generative AI	• Literature mining for novel targets • Protein structure prediction • Senolytic agent rational design	• Low efficiency of drug discovery • High attrition of traditional pipelines • Difficulty identifying novel therapeutic targets	Ansari et al. (2025), Ferro et al. (2025)
Patient digital twins	• Patient-specific virtual modeling • Immune-aging trajectory simulation • Personalized intervention prediction	• Static cross-sectional model limitations • Inability to capture longitudinal immune aging • Lack of individualized clinical prediction	Ferro et al. (2025)

First, advances in explainable AI (XAI), including SHAP, LIME, and attention-based transformer models, represent a critical breakthrough for addressing the “black box” nature of current AI models in immunosenescence research. These techniques enable researchers to decode the specific biological mechanisms underlying model predictions, enhancing causal interpretability of AI findings and building greater clinician trust in AI-driven clinical decision-making by providing transparent, justifiable rationale for model outputs (Chen et al., 2023). For XAI to have real impact, however, it must be integrated into model development from the start, rather than added as an afterthought to a pre-trained model.

In parallel, federated learning offers a robust framework to resolve the dual challenges of data privacy and cohort homogeneity that have long limited model generalizability. This distributed learning paradigm enables collaborative AI model training across multiple clinical and research institutions without sharing raw patient data, fully complying with global data protection regulations while unlocking access to large, diverse multi-center datasets that can reduce model bias and improve predictive performance across heterogeneous patient populations (Ma et al., 2025). Federated learning is particularly well-suited to immunosenescence research, where multi-center data sharing has been limited by privacy regulations and administrative barriers.

Furthermore, generative AI technologies—including large language models (LLMs) and generative deep learning architectures—have the potential to accelerate immunosenescence research and therapeutic development, directly addressing the inefficiency of traditional drug discovery pipelines. These models can systematically analyze the vast body of published scientific literature to identify unrecognized biological pathways and novel therapeutic targets for immunosenescence, accurately predict protein structures relevant to immune aging, and rationally design novel senolytic agents and immunomodulatory therapies, reducing the high attrition rates of traditional drug discovery pipelines (Ansari et al., 2025; Ferro et al., 2025). It is critical to note, however, that these tools remain adjuncts to experimental validation, not replacements: their outputs require rigorous *in vitro* and *in vivo* testing to confirm biological relevance.

Finally, patient digital twin technology complements these AI advances by enabling creation of virtual, patient-specific computational models that integrate multi-omics, clinical, and longitudinal health data to simulate individual immune aging trajectories and treatment responses. This technology overcomes the limitations of static cross-sectional AI models, capturing the dynamic, longitudinal nature of immunosenescence and enabling development of truly personalized, predictive interventions for immunosenescence-related diseases (Ferro et al., 2025). While this technology is still in its infancy, it offers a path to move beyond one-size-fits-all interventions for immune aging to truly personalized geriatric care.

### 6.2 Key future research trends

Driven by technological advances and the urgent unmet clinical needs of the global aging population, four interconnected research trends will shape the future of AI and ML applications in immunosenescence research, all centering on addressing the core gaps identified in this review.

The first dominant trend is the seamless multi-modal integration of high-dimensional biological and clinical data, a critical advancement given the complex, multi-layered nature of immunosenescence. Future AI models will move beyond single-data-type analyses to integrate information from single-cell and spatial transcriptomics, proteomics, metabolomics, medical imaging, wearable device metrics, and longitudinal EHRs, creating a comprehensive, multi-dimensional view of immune aging that captures the full complexity of the process and enables far more accurate prediction of clinical outcomes and treatment responses (Zhang et al., 2024). Critically, these models will be built with biological constraints, ensuring multi-modal integration aligns with known immunological principles, rather than just statistical correlations.

Methodologically, such integration can be implemented at several levels. Early fusion concatenates harmonized features across genomics, epigenomics, proteomics, metabolomics, microbiome data, and EHR variables, but is sensitive to missingness and batch effects. Late fusion combines modality-specific predictions and may be more robust in clinical settings where not every patient has every assay. Intermediate fusion methods, including autoencoders, latent-factor models, essential regression, and Bayesian multi-omics models, are better suited to building holistic immune-aging signatures while preserving uncertainty and prior biological knowledge (Bing et al., 2022). Graph neural networks add another layer by representing immune-cell subsets, cytokines, metabolites, microbial taxa, and clinical phenotypes as interacting nodes rather than isolated variables; in spatial datasets, related GNN approaches can also model tissue-neighborhood effects in aged immune microenvironments (Hu et al., 2021; Dong and Zhang, 2022; Ma et al., 2024). These integrative frameworks are important because clinical translation requires not only biomarker discovery, but also calibrated, interpretable signatures that remain usable when real-world data are incomplete.

Spatial transcriptomics is especially relevant because immune aging is not only a change in circulating cells but also a remodeling of tissue niches. Recent spatial transcriptomic studies of aging tissues have begun to localize inflammatory and senescence-associated programs *in situ*, while graph neural network (GNN) methods such as SpaGCN and STAGATE can integrate gene expression, spatial coordinates, and histology to infer spatial domains and cell-neighborhood relationships (Hu et al., 2021; Dong and Zhang, 2022; Ma et al., 2024). In immunosenescence, these approaches could help distinguish systemic immune aging from tissue-specific aged immune microenvironments, for example, by mapping macrophage, microglial, T-cell, and stromal interactions around senescent niches. At present, however, this remains a forward-looking direction: most spatial-GNN studies are methodological or disease-context studies rather than validated immune-aging clinical tools.

A related frontier is AI modeling of the microbiota-immune-aging axis. Microbiome data are compositional, sparse, and strongly confounded by diet, medication exposure, frailty, geography, and comorbidity, so simple taxon-by-taxon association testing is unlikely to identify clinically reliable drivers of immune aging. Future models should integrate metagenomic profiles, microbial metabolites, cytokine panels, immune-cell states, and longitudinal clinical records, using causal inference, longitudinal Bayesian models, or graph-based representations to distinguish microbial drivers from downstream consequences of systemic aging (Pallikkuth et al., 2021; DeJong et al., 2020; Bing et al., 2022; Haran et al., 2019). The most useful tools will capture dynamic crosstalk among microbiota composition, barrier function, immune-cell activity, inflammaging, and systemic aging phenotypes, rather than treating the gut microbiome as a stand-alone biomarker layer.

Closely linked to this is the second trend: the shift from static cross-sectional predictions to longitudinal dynamic modeling of immune aging trajectories. Most existing AI models in the field are trained on single time-point data, which cannot capture the progressive, dynamic nature of immunosenescence over the lifespan; future models will leverage long-term longitudinal cohort data to track immune aging changes over time, enabling early detection of accelerated immune aging and implementation of preclinical interventions to prevent age-related disease onset before clinical symptoms emerge (Zhang et al., 2024). This will require a shift in the field away from cross-sectional cohort studies to large, long-term longitudinal studies of immune aging in diverse populations.

The third critical trend is a dedicated focus on addressing health disparities in immunosenescence research and AI model development—an ethical and clinical priority given the uneven burden of age-related diseases across populations. Future research will prioritize creation of AI models trained on large, diverse, multi-ethnic cohorts, with specific attention to inclusion of underrepresented populations historically excluded from aging research, including frail older adults, racial and ethnic minorities, and individuals with multiple chronic comorbidities. This focus will reduce inherent model bias, improve generalizability across all patient populations, and advance health equity in geriatric care and immune aging interventions (Pinto et al., 2025).

Finally, the field will move towards development of mechanism-informed AI models. These models integrate well-established prior biological knowledge of immune system regulation and aging mechanisms directly into model architecture, shifting the field away from purely data-driven models (which often produce biologically implausible findings) towards biologically constrained models that enhance causal inference, improve interpretability, and ensure AI outputs align with fundamental principles of immunology and geroscience (Bing et al., 2022). Critically, these models will use biologically relevant endpoints (e.g., biological age, clinical outcomes, vaccine response) rather than chronological age as the training ground truth, addressing the fundamental flaw of many current models.

### 6.3 Clinical translation roadmap

To translate promising AI-driven advances in immunosenescence research into tangible clinical practice and improved patient outcomes, a structured, iterative clinical translation framework is required—one that abandons the idealized linear pipelines prevalent in most reviews and instead embraces the inherent unpredictability of translational research, where iterative refinement between preclinical and clinical work is essential, and many candidate biomarkers or tools will inevitably fail at various stages. This framework centers on addressing the specific barriers to real-world implementation outlined earlier, integrating the core requirements of each translational phase into a cohesive process rather than discrete steps. Rigorous preclinical validation forms the foundational prerequisite: AI-identified biomarkers and therapeutic targets must undergo comprehensive experimental validation in both *in vitro* systems and well-characterized *in vivo* animal models to confirm their biological relevance and causal role in immunosenescence, following the paradigm established by the preclinical validation of the iAge clock’s core mediator CXCL9 (Sayed et al., 2021), while also including technical validation in independent, held-out datasets to rule out overfitting or batch effects—only biologically meaningful and technically robust findings should proceed further.

Following successful preclinical validation, candidate tools must advance to large-scale, geographically and demographically diverse multi-center clinical validation, directly addressing the bias of current research toward single-center, homogeneous cohorts. These cohorts must intentionally include historically excluded groups—frail older adults, racial and ethnic minorities, and individuals with multiple chronic comorbidities—to ensure generalizability and equitable performance across the heterogeneous patient populations that will benefit most. The most critical step in this framework is prospective randomized controlled trials, a frequently skipped stage that is the only way to definitively prove AI-guided interventions improve patient-relevant clinical outcomes, rather than merely predicting them; examples include trials randomizing older adults to AI-guided vaccine stratification or personalized biologic therapy selection to demonstrate tangible benefits like improved vaccine efficacy or reduced adverse events, which is essential for regulatory approval and widespread adoption.

Two pragmatic first-step prospective studies would be feasible. First, a multi-center trial in adults aged 65 years or older presenting for seasonal influenza, COVID-19, or respiratory syncytial virus (RSV) vaccination could compare usual age-based vaccine selection with AI-guided stratification using a pre-vaccination immune-aging score. The primary endpoint could be seroconversion or neutralizing antibody response at 28–42 days, with secondary endpoints including cellular recall response, breakthrough infection over one season, adverse events, calibration across sex and comorbidity strata, and workflow time in primary-care or community vaccination clinics. Second, a frailty-prevention study could enroll community-dwelling, non-frail adults aged 70 years or older, perform baseline immune-aging assessment, and randomize those with accelerated immune age to a targeted intervention bundle, such as nutrition optimization, resistance exercise, medication review, and a validated senotherapeutic candidate when available, *versus* usual care. The primary endpoint would be incident frailty at 24 months; secondary endpoints would include infection frequency, hospitalization, and functional decline. These designs are more realistic than immediate broad deployment because they test whether the model changes decisions and improves outcomes in the setting where it would actually be used.

Building on positive trial outcomes, the framework progresses to regulatory approval and sustainable clinical implementation, requiring close collaboration between developers and global regulatory agencies to comply with AI/ML-based software as a medical device (SaMD) regulations, including comprehensive pre-market validation and robust post-market surveillance. Parallel investments in healthcare digital infrastructure and clinician training are also vital to ensure seamless integration of AI tools into routine workflows, as even rigorously validated tools will fail to benefit patients if they cannot be easily adopted or trusted by frontline providers. Finally, long-term post-market monitoring is an ongoing component of this framework, establishing standardized surveillance systems to track real-world performance, detect model drift or emerging bias, and refine tools based on real-world data—addressing the unique regulatory challenges of adaptive AI models and ensuring sustained safety, efficacy, and equity over time.

## 7 Conclusion

Immunosenescence is a central driver of age-related morbidity and mortality worldwide, with profound clinical implications for the rapidly growing global aging population. For decades, traditional research methodologies have been unable to fully decode the complexity and heterogeneity of immune aging, limiting the development of robust biomarkers and effective interventions that reach patient bedsides. AI and ML have emerged as promising tools that address these long-standing bottlenecks, enabling notable advances in mechanistic understanding, biomarker discovery, diagnostic innovation, and therapeutic development for immunosenescence.

In this review, we have systematically synthesized the state-of-the-art applications of AI/ML across the full translational spectrum of immunosenescence research, from basic mechanistic insights to clinical implementation. We first established the core biological and clinical foundation of immunosenescence, and outlined the critical limitations of traditional research methodologies that create a use case for AI/ML-driven approaches. We then broke down core ML methodologies relevant to immune aging research, and mapped key milestones in their integration with immunosenescence research over the past decade. We next comprehensively reviewed current applications and translational evidence of AI/ML in mechanistic discovery, biomarker development, diagnostic innovation, and therapeutic development for immunosenescence, highlighting both successes and the widespread reproducibility crisis in the field. We further critically analyzed the technical, ethical, and regulatory barriers that limit clinical translation of these advances, including the underrecognized, fundamental flaw of using chronological age as a model training endpoint, and provided a realistic, iterative roadmap for future research and clinical implementation.

While significant challenges remain, the integration of emerging AI technologies with immunosenescence research holds real potential to transform our understanding of immune aging and translate basic research findings into clinical interventions that promote healthy aging and reduce the burden of age-related diseases. Realizing this potential will require a fundamental shift in the field: away from “AI for AI’s sake” research that prioritizes model performance metrics over clinical utility, and towards rigorous, biologically grounded, multi-center research that prioritizes reproducibility, interpretability, and health equity. It will also require interdisciplinary collaboration between immunologists, geroscientists, AI researchers, clinicians, regulatory agencies, and patient advocates—groups that have historically worked in silos.

Looking ahead, the greatest value of AI in immunosenescence research will not come from ever-more complex deep learning models, but from simple, interpretable, biologically constrained tools that can be integrated into routine clinical workflows to identify at-risk older adults before the onset of age-related disease. The goal of this research should not be to build the most accurate model of chronological age, but to develop tools that meaningfully improve the health and quality of life of older adults. By addressing current limitations in model generalizability, interpretability, data diversity, and clinical validation, the field can move towards a future where AI-driven, personalized interventions for immunosenescence are routinely integrated into clinical care, improving health outcomes and quality of life for older adults worldwide.

## Glossary

AD: Alzheimer’s disease
AI: Artificial intelligence
AUC: Area Under the Curve
CHIP: clonal hematopoiesis of indeterminate potential
CNNs: Convolutional neural networks
EHRs: electronic health records
EORA: elderly-onset rheumatoid arthritis
ER: essential regression
FDA: Food and Drug Administration
GDPR: General Data Protection Regulation
GNN: graph neural network
HIPAA: Health Insurance Portability and Accountability Act
iAge: inflammatory aging clock
ISI: immunosenescence index
LASSO: least absolute shrinkage and selection operator
LIME: Local Interpretable Model-Agnostic Explanations
LLMs: large language models
MAE: mean absolute error
MDRO: multidrug-resistant organism
ML: machine learning
NSCLC: non-small cell lung cancer
PCA: principal component analysis
PD: Parkinson’s disease
PLS-DA: partial least squares-discriminant analysis
PMA: Pre-Market Approval
pQTL: protein quantitative trait loci
PTSD: post-traumatic stress disorder
RNNs: recurrent neural networks
SaMD: software as a medical device
SASP: senescence-associated secretory phenotype
scRNA-seq: single-cell RNA sequencing
SHAP: SHapley Additive exPlanations
SKCM: skin cutaneous melanoma
SVM: support vector machines
TCR: T cell receptor
XAI: explainable AI
